# Association between Multimorbidity and Suicidal Behaviors among People with Psychotic Disorders

**DOI:** 10.1093/schbul/sbag089

**Published:** 2026-10-30

**Authors:** Alice Saito, Florence Piché, Katerina Kavalidou, Aurelie Baillot, Robert-Paul Juster, Signature Consortium, Ahmed J Romain

**Affiliations:** School of Kinesiology and Physical Activity Sciences, Université de Montréal, Montreal, Quebec, H3T 1J4, Canada; Centre de Recherche de l’Institut Universitaire en Santé Mentale de Montréal, Montréal, Quebec, H1N 3V2, Canada; School of Kinesiology and Physical Activity Sciences, Université de Montréal, Montreal, Quebec, H3T 1J4, Canada; Centre de Recherche de l’Institut Universitaire en Santé Mentale de Montréal, Montréal, Quebec, H1N 3V2, Canada; National Suicide Research Foundation, University College Cork, Cork, T12 XF62, Ireland; Interdisciplinary School of Health, University du Québec en Outaouais, Gatineau, Quebec, J8X 3X7, Canada; Institut du savoir de l’Hôpital Montfort, Ottawa, Ontario, K1K 0T2, Canada; Centre de recherche du Centre Intégré de Santé et Services Sociaux de l’Outaouais, Gatineau, J8V 3R2, Quebec, Canada; Centre de Recherche de l’Institut Universitaire en Santé Mentale de Montréal, Montréal, Quebec, H1N 3V2, Canada; Center on Sex*Gender, Allostasis, and Resilience (CESAR), Montreal, Quebec, H1N 3V2, Canada; Department of Psychiatry & Addiction, University of Montreal, Montreal, Quebec, H3T 1J4, Canada; Centre de Recherche de l’Institut Universitaire en Santé Mentale de Montréal, Montréal, Quebec, H1N 3V2, Canada; School of Kinesiology and Physical Activity Sciences, Université de Montréal, Montreal, Quebec, H3T 1J4, Canada; Centre de Recherche de l’Institut Universitaire en Santé Mentale de Montréal, Montréal, Quebec, H1N 3V2, Canada

**Keywords:** psychotic disorders, suicidal ideations, suicidal risk, multimorbidity, physical health

## Abstract

**Background and Hypothesis:**

Suicide and poor health conditions are the leading causes of premature mortality in psychotic disorders. People with psychotic disorders have an increased risk of multimorbidity (≥2 chronic conditions) which could contribute to higher rates of suicidal ideation and suicide behaviors. The aim of the present study was to determine the association between multimorbidity and suicidal behaviors in people with psychotic disorders.

**Study Design:**

Cross-sectional study using data from the *Signature Biobank* and including people with clinical diagnoses of psychotic disorders. Sociodemographic information, lifestyle habits, 22 chronic conditions, suicidal ideation (PHQ-9), and suicidal behaviors (SBQ-R) scores were gathered. Logistic regressions were performed across three models with different levels of adjustment: sociodemographics (M1), M1 adjusted for psychotic symptoms (M2), and M2 with further adjustments for lifestyle habits (M3). Sensitivity analyses were done on a subgroup of individuals with early psychosis (≤30 years old).

**Study Results:**

A sample of 748 participants with diagnosis of psychotic disorder (69% male; age: 39.5 ± 14.1; 35.2% with schizophrenia was recruited. Among them, 407 participants (54%) had multimorbidity, and 44% had physical multimorbidity. After adjustment for covariates, individuals with multimorbidity were more likely to report suicidal ideations (OR 2.31, 95%CI [1.61–3.32]) and suicidal behaviors (OR 1.84, 95%CI [1.15–2.97]) than their counterparts. Similar results were found for the young psychosis subgroup (<30 years old), as for physical multimorbidity.

**Conclusion:**

Results confirmed an association between multimorbidity and higher rates of suicidal behaviors among our sample, supporting the importance of interventions to prevent the development of chronic conditions.

## Introduction

Individuals with a psychotic disorder have a reduced life expectancy of 10 to 25 years compared to the general population, with cardiovascular diseases and suicide being the leading causes of premature death.^1^ The lifetime prevalence of suicidal ideation is 35% of individuals with a schizophrenia spectrum disorder, compared to ~9% in the general population,^2^ and their estimated lifetime risk of death by suicide ranges from 4% to 13%.^3^ These elevated rates are alarming, especially when considering that suicidal ideation is a risk factor for suicide attempts and subsequent suicide deaths.^4^ Moreover, ~60% of suicide attempts occur among people who previously experienced ideations or engaged in self-injurious behaviors within the preceding three months.^2^^,^^5^

Suicidal risk comprises factors that may lead to suicidal ideation, self-harm, and for some people, to attempt or death by suicide.^6^ Establishing why individuals with psychotic disorders present increased suicidal behaviors and risks is complex and likely influenced by multiple interacting variables such as sociodemographic (eg, sex, age) and lifestyle factors (eg, substance use). Regarding sociodemographic, individuals with psychotic disorders often have lower education, socioeconomic status, and higher unemployment, rates than the general population,^7^ which are risks factors for suicidal behaviors.^8^

Besides, positive psychotic symptoms (eg, hallucination) have been associated with a higher prevalence of suicidal behaviors and risk while negative symptoms (eg, amotivation) do not show a consistent association with suicidal ideation, and may even lead to a reduction of death by suicide.^9^ In addition, age has been associated with variation in suicidal behaviors, with the strongest correlation observed among those under 45 years old.^3^ However, it is important to highlight that the onset of psychotic disorder usually happens between 20 and 30 years old, and this association may be linked with illness onset, rather than age alone. This hypothesis is reinforced when considering that the highest suicide risk exists during the first decade following diagnosis.^3^

Regarding lifestyle factors, substance use is common in populations with psychotic disorders with 40%–70% of individuals with schizophrenia experiencing alcohol or non-prescribed drug dependence, and ˃70% are dependent on nicotine.^7^^,^^10^ Substance use represents a significant suicide risk, as alcohol and drug use disorders are associated with higher rates of suicidal ideations, suicidal behaviors, and death by suicide.^11^

Finally, while many suicide risk factors overlap with those in the broader population, factors like fear of cognitive decline, restlessness, and poor treatment adherence are more specific to individuals with psychotic disorders.^12^ Furthermore, males and inpatients are generally at higher risks of suicide attempts, possibly due to increased stigma, unemployment, more frequent alcohol and substance use, greater impulsivity, and higher rates of aggression among males compared to females.^2^^,^^13^

Among other factors, the impact of physical health, notably chronic conditions, on suicidal ideation and behaviors has received limited attention. Previous studies showed that the accumulation of chronic conditions has been associated with increased risks of suicidal ideation, particularly in the general population, but also in people with obesity, or various mental health disorders (eg, anxiety and mood disorders).^14^^,^^15^ This accumulation of chronic conditions is known as multimorbidity and is defined as the co-occurrence of at least two chronic conditions, such as physical and mental health conditions, or infectious disease of long duration.^16^^,^^17^ A previous meta-analysis showed that people with psychotic disorders are approximately three times more likely to have multimorbidity than people without psychotic disorders.^18^^,^^19^ Multimorbidity has been found to be associated with higher hospitalization and use of health services in people with severe mental illness^20^ but its specific impact on suicidal behaviors in people with psychotic disorders has not been investigated. Moreover, the specific impact of physical conditions on suicidal behaviors has not been examined.

Considering that individuals with psychotic disorders are more likely to experience suicidal behaviors, and to have multimorbidity, and that these two factors are leading causes of premature mortality,^1^ that multimorbidity is associated with increased suicide risk in other populations, it was necessary to study these two factors together in this specific group.^14^^,^^15^^,^^19^ Thus, the aim of the present study was to explore the association between multimorbidity and suicidal behaviors among people with psychotic disorders. It is hypothesized that individuals with psychotic disorders and multimorbidity will be more likely to report suicidal ideation and suicidal behaviors.

## Methods

The present study was approved by the ethics committee of the CIUSSS de l’Est-de-l’île-de-Montréal with the number # 2022-2731.

### Procedure and Settings

The present cross-sectional study used data from the *Signature Biobank* (https://www.banquesignature.ca) based at the Centre de recherche de l’Institut universitaire de santé mentale de Montréal (Québec, Canada). This biobank is a large repository of patients’ biospecimens (n = 1986), psychosocial questionnaires (n = 2085), sociodemographic, and diagnostic data collected during and after their visit to the psychiatric emergency department between 2012 and 2020.^21^

### Study-Specific Inclusion Criteria

Participants were eligible if they were over 18 years old and had a formal diagnosis of psychotic disorder by a psychiatrist, see^21^ for more details)*.*

### Measurements

#### Suicidal Ideation and Suicidal Risks

The *Patient-Health Questionnaire-9* (PHQ-9) was used to assess the presence of suicidal ideation. This questionnaire evaluates the severity of nine symptoms of depression based on the DSM-IV.^22^ Respondents were asked to self-report and rate each item on a scale from “0” (not at all) to “3” (nearly every day).^23^ For the present study, we used item 9 of the PHQ-9 (“Over the last 2 weeks, how often have you been bothered with the following problems: thoughts that you would be better off dead, or of hurting yourself in some way?”),^23^ as it directly measured the presence of suicidal ideations. Participants were asked whether they had experienced suicidal ideation, indicating the frequency as “never,” “several days,” “more than 7 days”, or “almost every day”. Participants who responded “several days,” “more than 7 days,” or “almost every day,” were considered to have suicidal ideation. To run sensitivity analyses, we also considered suicidal ideation based on the continuous score. The PHQ-9 has been validated for both depression diagnosis severity assessment and the screening of suicidal ideation.^23^

To evaluate suicidal behaviors, we used the *Suicidal Behavior Questionnaire-Revised* (SBQ-R*)*, a four-item self-report questionnaire which aims to identify the presence of suicidal ideations, suicidal behaviors, as well as past, and possibly future suicide attempts, while quantifying their frequency throughout the past year. The *SBQ-R* has been shown to have high reliability and significant moderate validity compared to other similar questionnaires. A score of ≥8 points on 18 on the questionnaire means a risk of suicidal behaviors.^24^

In our study, we used the PHQ-9 instead of the SBQ-R as main outcome, because the SBQ-R was added 5 years after the beginning of data collection in the Signature Biobank (2017). Therefore, SBQ-R data was not available for all 748 participants. However, including this questionnaire still allowed secondary analyses of suicidal behaviors in a subgroup of participants, complementing our main analyses on suicidal ideation with the PHQ-9.

#### Multimorbidity

In the present study, multimorbidity was defined as two or more co-occurring somatic and/or psychiatric conditions in the same individual. Multimorbidity was measured using a module from the Canadian Community Health Survey during the participant’s screening at the emergency room of the *Institut universitaire en santé mentale de Montréal.* Data was collected on 23 chronic conditions: asthma, arthritis, back pain, hypertension, migraines, early bronchitis, emphysema, cardiopathies, ulcers, strokes, cancer, incontinence, digestive disorders, type 2 diabetes, Alzheimer, Chronic obstructive pulmonary disease (COPD), obesity, triglycerides, epilepsy, mood disorders, anxiety disorders, personality disorders, as well as substance use disorders. To be considered as chronic, the condition had to last or be expected to last at least 6 months. Except obesity, hypertension, and psychiatric disorders which were evaluated by a health professional, all other conditions were self-reported using a dichotomous response choice then discussed with a health professional.^21^ To specifically account for the contribution of physical conditions, we also computed a measure of physical multimorbidity which was the accumulation of two and more somatic conditions within the same individual.

#### Covariates

Collected covariates were age, sex, employment status, substance use (tobacco, alcohol, drugs), and psychotic symptoms.

Regarding tobacco use, all individuals were asked about smoking habits. Questions were taken from the Canadian Community Health Survey, and included the age at which the person had started smoking, whether they smoked daily, and if they had ever attempted to stop smoking in the past 12 months.^25^

To assess alcohol consumption, participants completed the Alcohol Use Disorders Identification Test questionnaire (AUDIT), a screening questionnaire that evaluates total intake, dependence symptoms, and alcohol-related harm.^26^ The AUDIT is a widely validated screening tool for alcohol use.^27^ The self-report version of the questionnaire includes 40 questions addressing various alcohol-related behaviors or situations, each scored from 0 to 4, ranging from “never” to “4 or more times a week”. Upon completion, respondents receive a score out of 40 points, with scores ≥8 representing harmful alcohol use and possible dependence.^28^ In the present study, analyses were conducted with continuous variables based on each participants’ score.

Drug use was measured using the Drug Abuse Screening Test (DAST-10), a self-report or interview screening-tool evaluating drug use, excluding alcohol and tobacco, during the past 12 months.^29^ The questionnaire categorizes individuals based on their score over 10 points: 0 (no problem), 1–2 points (low problems), 3–5 (moderate), 6–8 (substantial), and 9–10 (severe). The DAST-10 is an evidence-based measure recommended by national agencies and has shown high validity, reliability, as well as diagnostic sensitivity.^30^ In the present study, participants completed the self-report version of the DAST-10,^29^ after which they received their individual score based on the number of symptoms exhibited, therefore the analyses were conducted based on continuous variables.

Psychotic symptoms were assessed using the Psychosis Screening Questionnaire Short-Scale (PSQ-SS). This self-report tool examines the presence and severity of psychotic symptoms during the past year. The questionnaire consists of five questions, each about a specific symptom (hypomania, thought insertion, paranoia, strange experience, as well as hallucinations), with follow-up questions when a symptom is endorsed.^31^ For analyses, the presence of psychotic symptoms was measured continuously based on the total number of reported symptoms, on a scale of 0–5.

### Statistical Analyses

To evaluate the association between suicidal ideation and multimorbidity, we used logistic regression analyses, and results are odds ratio with 95% confidence intervals (95%CI). To account for different covariates, three separate models were performed for the analysis of suicidal ideation as per PHQ-9, SBQ-R and suicidal behaviors overall (SBQ-R). The three models were as follows: Model 1 (M1) was adjusted for age, sex, and employment status. Model 2 (M2) included the same covariates as M1, with the addition of psychotic symptoms. Model 3 (M3) included the same covariates as M2 and further included alcohol and drug use as additional covariates. The same analyses were performed with physical multimorbidity.

Similarly to primary analyses, sensitivity analyses were conducted to assess the robustness of the results based on a subgroup with specific features in terms of suicidal ideation risk. These were run using the same three models (M1, M2, and M3) as for primary analyses, but among participants under 30 years old with psychosis. In this sensitivity analysis, the diagnosis of alzheimer has been removed from the list of conditions used to compute multimorbidity.

To examine a potential dose–response relationship between multimorbidity and suicidal ideation, a restricted cubic spline model was performed and adjusted for covariates. The figure displays the estimated association across increasing levels of multimorbidity.

Statistical analyses were performed using RStudio (version 2024.12.0 + 467) and the packages *summarytools,*^32^  *dplyr,*^33^  *tidyverse,*^34^  *psych,*^35^  *ISLR,*^36^  *pscl,*^37^  *caret,*^38^  *car,*^39^  *gtsummary,*^40^  *rcs, and finalfit.*^41^ Statistical significance was established using a *P*-value ≤.05. Codes used for analyses are in Supplementary Files.

## Results

### Sample Characteristics

The present cross-sectional analysis included 748 participants among which 704 (94.1%) were diagnosed with a psychotic disorder, and 44 (5.9%) had a mood disorder with psychotic features. Among those with psychotic disorders, the main diagnoses (reported as per ICD-11) were schizophrenia (n = 263, 35.2%), schizophrenia-like psychotic disorder (n = 263, 35.2%), and schizo-affective disorder (n = 132, 17.6%). Most participants were males (n = 519; 69.4%) with a mean age of 39.5 ± 14.1 years old. Regarding educational attainment, 277 participants (37.0%) had not completed high school, while 466 (62.3%) had at least a high school diploma. Additionally, 578 participants (77.5%) were unemployed at the time of the study. For housing situations, the majority were living in private residences (n = 535; 71.5%). Regarding lifestyle behaviors, 344 participants (46.0%) were daily smokers, 271 (36.2%) had a moderate to severe risk related to drug use based on the DAST-10 questionnaire, and 159 (21.3%) with harmful alcohol use or dependence according to the AUDIT questionnaire (Table 1).

**Table 1:** Sociodemographic Characteristics of Participants (N = 748).

	Primary analysis (N = 748)	Secondary analysis (<30 y.o.) (N = 240)
**Sex N, %**		
**Male**	519 (69.4)	180 (75.0)
**Female**	229 (30.6)	60 (25.0)
**Employment Status N, %**		
**Employed**	169 (22.5)	66 (27.5)
**Unemployed**	578 (77.5)	174 (72.5)
**Education Achievement N, %**		
**No high school diploma**	273 (36.5)	117 (48.8)
**High school diploma**	192 (25.7)	54 (22.5)
**College or Certificate**	140 (18.7)	36 (15.0)
**University diploma**	39 (5.2)	9 (3.8)
**NA**	104 (13.9)	24 (10.0)
**Living situation N, %**		
**Private housing**	535 (71.5)	186 (77.5)
**Group housing (>10 people)**	43 (5.8)	8 (3.3)
**Group housing (<10 people)**	21 (2.8)	–
**Social housing**	91 (12.3)	7 (2.9)
**No permanent housing/homeless**	18 (2.4)	7 (2.9)
**Others** ^**a**^	40 (5.4)	32 (13.3%)
**Primary Diagnosis N, %**		
**Psychotic disorder**	704 (94.1)	226 (94.2)
**Mood disorder**	44 (5.9)	14 (5.8)
**Multimorbidity (≥2 conditions)**	407 (54.4)	114 (47.5)
**Physical multimorbidity (≥2 conditions)**	332 (44.4)	107 (44.6)

a Includes long-term hospitalization, forensic unit, prison, and missing data.

In the present sample, the prevalence of multimorbidity was 54.4% (n = 407), and 44.4% for physical multimorbidity (n = 332). The most prevalent conditions were back pain (n = 244, 32.6%), migraines (n = 195, 26.1%), hypertension (n = 105, 14.0%), mood disorders (n = 259, 34.6%), and anxiety disorders (n = 244, 32.6%) (prevalences are in Supplementary Material 1).

The top 5 dyads were mood and anxiety disorders (n = 155, 20.7%), back pain and mood disorder (n = 114, 15.2%), back pain and anxiety disorder (n = 113, 15.1%), back pain and migraine (n = 109, 14.5%), followed by migraine and mood disorder (n = 101, 13.5%).

### Association between Suicidal Ideation and Multimorbidity

In the present study, the prevalence of suicidal ideation was 32.4% (n = 242) based on participants’ answers to item 9 of the PHQ-9.

Regarding the association between suicidal ideation and multimorbidity in M1, participants with multimorbidity were three times more likely to experience suicidal ideations (OR: 3.00, 95%CI [2.14–4.23]; *P* < .001). Among covariates, age was negatively associated with suicidal ideation (OR: 0.99; 95%CI [0.98-1.00]; *P* = .05). No associations were found with sex (*P* = .5) or unemployment (*P* = .6) (Table 2).

**Table 2:** Results of the Association between Multimorbidity and Suicidal Ideation – (PHQ-9).

(N = 748)	OR	CI 95%	*P*-value
**Model 1**			
**Multimorbidity**	3.00	2.14–4.23	<.001
**Age**	0.99	0.98–1.00	.05^*^
**Sex**	1.13	0.80–1.61	.5
**Unemployed**	1.11	0.76–1.63	.6
**Model 2**			
**Multimorbidity**	2.60	1.84–3.72	<.001
**Age**	0.99	0.98–1.00	.04
**Sex**	1.17	0.82–1.68	.4
**Unemployed**	1.05	0.71–1.56	.8
**Psychotic symptoms**	1.35	1.19–1.53	<.001
**Model 3**			
**Multimorbidity**	2.31	1.61–3.32	<.001
**Age**	0.99	0.98–1.01	.4
**Sex**	0.98	0.68–1.43	>.9
**Unemployed**	1.07	0.72–1.60	.8
**Psychotic symptoms**	1.33	1.17–1.51	<.001
**AUDIT**	1.04	1.01–1.06	.002
**DAST-10**	1.04	0.97–1.12	.3

Model 1: adjusted for sociodemographic measures, Model 2: adjusted for psychotic symptoms, Model 3: adjusted for psychotic symptoms and lifestyle factors, PSQ-SS: Psychotic Screening Questionnaire, PHQ9: Patient Health Questionnaire-9, SBQ-R: The Suicide Behaviors Questionnaire-Revised AUDIT: Alcohol Use Identification Test, DAST-10: Drug Abuse Screening Test; OR: Odds ratio.

After adjusting for psychotic symptoms (M2), individuals with multimorbidity remained more than twice as likely to report suicidal ideation (OR: 2.60, 95%CI [1.84–3.72]; *P* < .001). Among covariates, psychotic symptoms (OR: 1.35; 95%CI [1.19–1.53]; *P* < .001), and age (OR: 0.99; 95%CI [0.98–1.00]; *P* = .04) were positively and negatively associated, respectively, with suicidal ideation (Table 2).

Finally, after adjusting for lifestyle factors (M3) multimorbidity remained significantly associated with suicidal ideation (OR: 2.31; 95%CI [1.61–3.32]; *P* < .001). Among covariates, alcohol consumption (OR: 1.04, 95%CI [1.01–1.06]; *P* = .002) and psychotic symptoms (OR: 1.33; 95%CI [1.17–1.51]; *P* < .001) were found to be positively associated as well (Table 2).

A visual summary of the results is provided in Figure 1.

**Figure 1:**
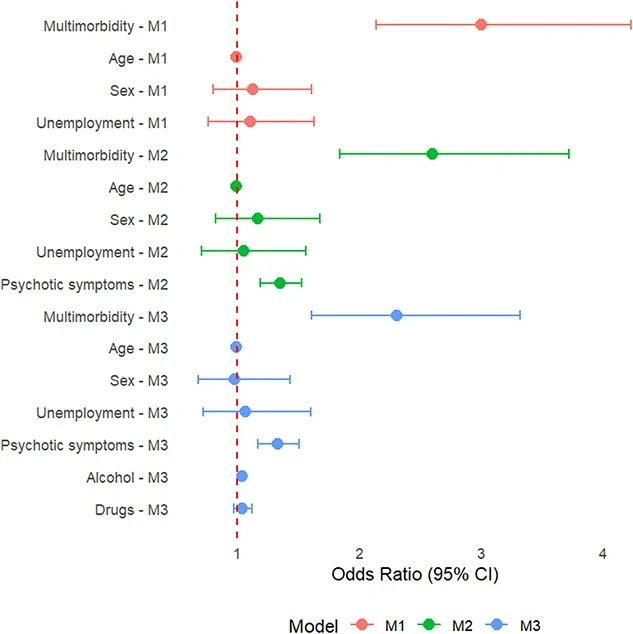
Association between Suicidal Ideation (PHQ-9) and Multimorbidity. Notes: M1: Model 1, M2: Model 2, M3: Model 3

Regarding physical multimorbidity, the same results were found and participants with physical multimorbidity were times more likely to experience suicidal ideations in M1 (OR: 2.15, 95%CI [1.51–3.06]; *P* < .001), M2 (OR: 2.11, 95%CI [1.47–3.05]; *P* < .001), and M3 (OR: 1.95, 95%CI [1.35–2.86]; *P* < .001) (see complete results in Supplementary File 2).

### Association between Suicidal Behaviors and Multimorbidity

Based on participants’ answers on the SBQ-R, the prevalence of suicidal risks was 30.1% (n = 132) among the sample.

Regarding the association between suicidal behaviors and multimorbidity (M1), individuals with multimorbidity were more than twice as likely to present a suicide risk (OR: 2.60; 95%CI [1.69–4.05]; *P* < .001). Among covariates, male sex (OR: 1.63; 95%CI [1.01–2.69]; *P* = .04), and age (OR: 0.98; 95%CI [0.97–1.00]; *P* = .05) were associated with suicidal behaviors (Table 3).

**Table 3:** Results of the Association between Multimorbidity and Risk of Suicidal Behaviors (SBQ-R).

N = 439	OR	CI 95%	*P*-value
**Model 1**			
**Multimorbidity**	2.60	1.69–4.05	<.001
**Age**	0.98	0.97–1.00	.04
**Sex**	1.63	1.01–2.69	.049
**Unemployed**	1.22	0.75–2.03	.4
**Model 2**			
**Multimorbidity**	2.20	1.40–3.48	<.001
**Age**	0.98	0.97–1.00	.042
**Sex**	1.76	1.07–2.97	.03
**Unemployed**	1.19	0.72–2.01	.5
**Psychotic symptoms**	1.47	1.24–1.74	<.001
**Model 3**			
**Multimorbidity**	1.84	1.15–2.97	.011
**Age**	0.99	0.98–1.01	.5
**Sex**	1.44	0.86–2.47	.2
**Unemployed**	1.19	0.71–2.03	.5
**Psychotic symptoms**	1.43	1.21–1.70	<.001
**AUDIT**	1.05	1.02–1.08	.003
**DAST-10**	1.07	0.97–1.18	.2

Model 1: adjusted for demographic measures, Model 2: adjusted for psychotic symptoms, Model 3: adjusted for psychotic symptoms and lifestyle factors, PSQ-SS: Psychotic Screening Questionnaire, PHQ9: Patient Health Questionnaire-9, SBQ-R: The Suicide Behaviors Questionnaire-Revised AUDIT: Alcohol Use Identification Test, DAST-10: Drug Abuse Screening Test; OR: odds ratio.

After adjusting for psychotic symptoms (M2), participants with multimorbidity remained twice as likely to have suicidal behaviors (OR: 2.20; 95%CI [1.40–3.48]; *P* < .001). Among covariates, psychotic symptoms (OR: 1.47; 95%CI [1.24–1.74]; *P* < .001) and sex (male) (OR: 1.76; 95%CI [1.07–2.97]; *P* = .03) were positively associated with risks of suicidal behaviors (Table 3), while age (OR: 0.98; 95%CI [0.97–1.00]; *P* = .04) was negatively associated.

In M3, after adjustment on lifestyle factors, individuals with multimorbidity were more likely to present suicidal behaviors (OR: 1.84; CI95% [1.15–2.97]; *P* = .01). Among covariates, psychotic symptoms (OR: 1.43; 95%CI [1.21–1.70]; *P* < .001) and alcohol consumption (OR: 1.05; 95% CI [1.02–1.08]; *P* = .003) were positively associated with increased suicidal behaviors (Table 3).

Regarding physical multimorbidity, the same results were found and participants with physical multimorbidity were times more likely to have suicidal behaviors in M1 (OR: 2.21, 95%CI [1.39–3.54]; *P* < .001), M2 (OR: 2.08, 95%CI [1.28–3.39]; *P* = .003), and M3 (OR: 1.86, 95%CI [1.13–3.08]; *P* = .016) (see complete results in Supplementary File 3).

A visual summary is provided in Figure 2.

**Figure 2:**
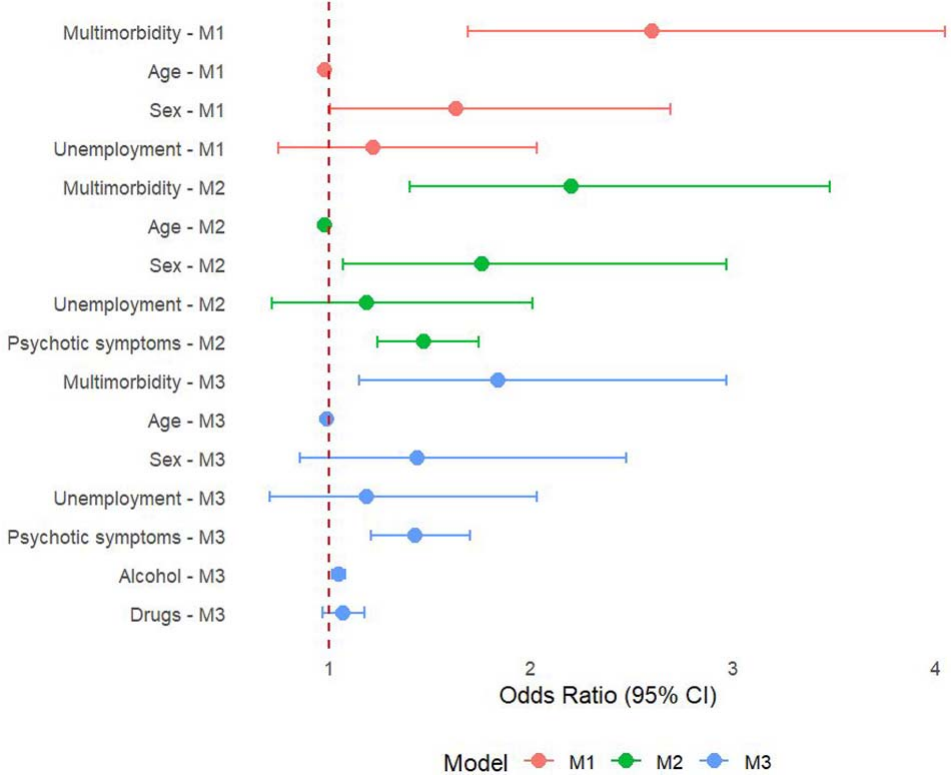
Association between Suicidal Behavior (SBQ-R) and Multimorbidity. Notes: M1: Model 1, M2: Model 2, M3: Model 3

### Sensitivity Analyses – Young Adults with Psychosis (<30 Years Old)

A sensitivity analysis (Table 4) was conducted with an early psychosis subgroup of participants (n = 240) under 30 years old who answered the PHQ9 and SBQ-R questionnaires. The subgroup had of majority of male participants (n = 180, 75%) with an average age of 24.2 ± 3.5 years old. Moreover, 44.6% (n = 107) of this subgroup had multimorbidity, and 30.4% (n = 73) had physical multimorbidity.

**Table 4:** Association between Multimorbidity and Suicidal Ideation in Young Adults (<30 Years Old) with Psychosis (PHQ-9).

	OR	CI 95%	*P*-value
**PHQ9-09**			
**Model 1**			
**Multimorbidity**	3.53	2.01–6.30	<.001
**Age**	0.98	0.90–1.06	.6
**Sex**	1.24	0.65–2.42	.5
**Unemployed**	1.18	0.63–2.25	.6
**Model 2**			
**Multimorbidity**	2.77	1.50–5.18	.001
**Age**	0.98	0.90–1.07	.7
**Sex**	1.45	0.73–3.01	.3
**Unemployed**	1.03	0.53–2.02	>.9
**Psychotic symptoms**	1.59	1.23–2.06	<.001
**Model 3**			
**Multimorbidity**	2.37	1.25–4.52	.008
**Age**	0.97	0.89–1.06	.6
**Sex**	1.29	0.63–2.70	.5
**Unemployed**	1.01	0.52–1.99	>.9
**Psychotic symptoms**	1.55	1.20–2.02	<.001
**AUDIT**	1.03	0.99–1.07	.2
**DAST-1**	1.06	0.95–1.19	.3
**SBQ-R**			
**Model 1**			
**Multimorbidity**	3.76	1.82–8.01	<.001
**Age**	0.98	0.88–1.09	.7
**Sex**	0.93	0.40–2.20	.9
**Unemployed**	1.36	0.62–3.10	.5
**Model 2**			
**Multimorbidity**	2.96	1.35–6.66	.008
**Age**	0.99	0.89–1.11	.9
**Sex**	1.06	0.43–2.73	.9
**Unemployed**	1.19	0.51–2.84	.7
**Psychotic symptoms**	1.54	1.10–2.20	.014
**Model 3**			
**Multimorbidity**	2.38	1.02–5.60	.045
**Age**	0.99	0.88–1.11	.9
**Sex**	0.94	0.37–2.47	>.9
**Unemployed**	1.21	0.51–2.96	.7
**Psychotic symptoms**	1.47	1.03–2.13	.035
**AUDIT**	1.05	1.00–1.11	.06
**DAST-10**	1.06	0.91–1.24	.5

Model 1: adjusted for demographic measures, Model 2: adjusted for psychotic symptoms, Model 3: adjusted for psychotic symptoms and lifestyle factors, PSQ-SS: Psychotic Screening Questionnaire, PHQ9: Patient Health Questionnaire-9, SBQ-R: The Suicide Behaviors Questionnaire-Revised AUDIT: Alcohol Use Identification Test, DAST-10: Drug Abuse Screening Test; OR: odds ratio.

#### Association between Suicidal Ideation and Multimorbidity (PHQ-9)

Among participants under 30 years old, 82 (34.17%) experienced suicidal ideation. Regarding M1, the likelihood of suicidal ideation was more than three times higher among those under 30 years old also presenting with multimorbidity (OR: 4.42; 95%CI [2.46–8.17]; *P* < .001) (Table 4). After adjusting for psychotic symptoms (M2), participants with multimorbidity remained more likely to have suicidal ideations (OR: 3.62; 95%CI [1.91–7.01]; *P* < .001). Among covariates, psychotic symptoms were also positively associated with suicidal ideations (OR:1.59; 95%CI [1.23–2.10]; *P* < .001) (Table 4).

In the third model (M3), participants with multimorbidity were twice more likely to have suicidal ideations (OR: 3.13; 95%CI 1.60–6.22; *P* < .001). Among covariates, only psychotic symptoms (OR: 1.56; 95%CI 1.20–2.06; *P* = .001) were positively associated with suicidal ideation (Table 4).

Regarding physical multimorbidity, consistent results were found and participants with physical multimorbidity were more likely to have suicidal behaviors in M1 (OR: 2.62, 95%CI [1.43–4.85]; *P* = .002), M2 (OR: 2.57, 95%CI [1.35–4.96]; *P* = .004), and M3 (OR: 2.33, 95%CI [1.19–.56]; *P* = .013) (see complete results in Supplementary File 4).

#### Association between Suicidal Behavior and Multimorbidity (PHQ-9)

Among the participants under 30 years old, 34.03% (n = 49) were considered at risk for suicidal behaviors. In M1, individuals with multimorbidity were over three times as likely to present a suicide risk (OR: 4.45; 95%CI [2.10–9.89], *P* < .001). No covariates were associated with increased risk (Table 4).

After controlling for psychotic symptoms (M2), multimorbidity remained significantly associated with increased risk of suicidal behaviors (OR: 3.68; 95%CI [1.64–8.62]; *P* = .002). Among covariates, the presence of psychotic symptoms was positively associated with increased risks of suicidal behaviors among this group (OR:1.64; 95%CI [1.16–2.38]; *P* = .01) (Table 4).

In M3, individuals with multimorbidity were more likely to present a risk of suicidal behaviors (OR:3.11; 95%CI [1.32–7.56]; *P* = .01). Among covariates, psychotic symptoms were associated with risk of suicidal behaviors (OR: 1.55; 95%CI [1.08–2.27]; *P* = .02). Other covariates were not significantly associated with an increased risk of suicidal behaviors in participants under 30 years old (Table 4).

Regarding physical multimorbidity, consistent results were found and participants with physical multimorbidity were more likely to have suicidal behaviors in M1 (OR: 3.02, 95%CI [1.38–6.78]; *P* = .006), M2 (OR: 2.84, 95%CI [1.22–6.71]; *P* = .016), and it was slightly significant in M3 (OR: 2.27, 95%CI [0.93–5.58]; *P* = .07) (see complete results in Supplementary File 5).

#### Dose-response Association between Suicidal Ideation, Behavior and Multimorbidity

The restricted cubic spline figure showed a non-linear dose-response association between suicidal ideation and multimorbidity with the steepest increase observed for the first few chronic conditions, followed by a plateau at higher levels of multimorbidity, suggesting that additional conditions continue to raise suicidal ideation but with diminishing incremental effect (Supplementary File 6). The same results were found for physical multimorbidity (Supplementary File 7). Regarding suicidal behavior, the same associations were found with multimorbidity and physical multimorbidity (Supplementary File 8, 9) with the steepest increase observed for the first few chronic conditions, but no plateau with higher levels of multimorbidity was observed suggesting that additional conditions contributing to suicidal behavior with an incremental effect.

## Discussion

The aim of the present study was to investigate whether multimorbidity was associated with suicidal ideation and risk of suicidal behaviors among individuals with a diagnosis of psychotic disorder. Briefly, we found that 54% of our sample had multimorbidity, which is higher than previous studies among this population.^18^^,^^19^ Additionally, a third of the sample (32.4%) reported experiencing suicidal ideations within the previous two weeks. This also corroborates previous findings reporting suicidal ideations within ~35% of individuals with a psychotic disorder.^2^

Regarding the association between multimorbidity and suicidal behaviors, we hypothesized that a positive association would be found, which was confirmed. In our study, multimorbidity was the variable most strongly associated with both suicidal ideations and risk of suicidal behaviors. In the first case, participants with multimorbidity were up to three times as likely to experience suicidal ideations than their counterparts who did not. This association remained significant in all three models independently of adjustment on covariates. Interestingly, it is also stable across our different secondary and sensitivity analyses (eg, young adults with psychosis, physical multimorbidity, dose–response association). These findings are consistent with previous studies showing associations between multimorbidity and suicidal behaviors in different populations, such as individuals with obesity or with common mental health disorders (eg, anxiety and mood disorders).^14^^,^^15^ Regarding the dose–response associations, the stronger association observed with suicidal behaviors compared to suicidal ideation may suggest a differential impact of multimorbidity across the spectrum of suicidality, which should be investigated in future studies.

In addition, we conducted sensitivity analyses on an early psychosis subgroup (under 30 years old). Similarly to previous research,^42^ the prevalence of multimorbidity was lower in the younger group, showing a slight increase with age. However, 48% of these participants did have co-occurring conditions, confirming a higher prevalence in individuals with early psychosis compared to their peers.^43^ Notably, higher rates of anxiety disorders (35.4% vs. 32.62%), as well as increased prevalence of suicidal ideation (34.1% vs. 32.4%) and suicide risk (34.0% vs. 30.1%) were observed in this younger subgroup compared to the general sample. This difference could be explained by onset of the disorder, which usually happens in the 20s and 30s, generally showing an increased suicide risk in younger individuals.^10^ This early psychosis group also showed higher drug use and lower alcohol use than the general sample, though these variables were not significantly associated with suicidal ideation or risks. Finally, psychotic symptoms were significantly associated with suicidal ideations and risks across all three models, corroborating findings from the general sample. However, odd ratios were higher in early psychosis group than in the general sample, indicating increased risk of suicidal behaviors when experiencing positive symptoms or chronic health conditions.

Among covariates, surprisingly no significant associations were found for variables normally related to suicidal ideations, such as sex, age, employment status, as well as drug use. While there was a small protective effect of age in M1 and M2, the association was no longer significant in M3, when adding lifestyle variables. These results, which conflict with previously published research,^2^^,^^3^^,^^10^ could be explained by the nature of the study’s recruitment strategy. In the *Signature Biobank*, participants were recruited from the emergency department at the *Institut universitaire de santé mentale*. Although specific entry motives were not disclosed, it is plausible that some participants were experiencing an acute crisis state, leading them to be admitted. Consequently, it could have influenced some variables’ outcomes, such as prevalence of substance use, as well as suicidal behaviors and suicidal thoughts. These results support the necessity to conduct similar analyses longitudinally to assess the effects of multimorbidity on suicidal ideations over a longer period, specifically following the participants’ discharge from the hospital as it is a known risk period for suicidal behaviors.^10^

Psychotic symptoms remained significantly associated with suicidal ideations aligning with previous findings showing a role of positive symptoms on suicidality,^44^ especially due to increased hopelessness, helplessness, and social isolation.^10^ Finally, a small association between alcohol use and suicidal ideations was found in M3, aligning with previous research. Indeed, the relation between alcohol and suicidal ideation has been previously established, as alcohol is shown to increase maladaptive coping, risk taking and impulsivity, as well as decrease efficient self-regulation, therefore leading to higher suicide risk.^11^

A major strength of this study came from the sample size which provided enough power to run our statistical analyses. Indeed, the *Signature Biobank* allowed the collection of a wide range of information on 2172 participants, among which our 748 participants with psychotic disorders were selected from, enabling the measurement of an extensive range of variables, as well as a more complete portrait of our sample. This significant sample also supported sensitivity analyses on participants with psychotic disorder under 30 years old, a subgroup particularly at risk of suicidal behaviors.^44^^,^^45^ Also, we had access to a large number of conditions to compute multimorbidity to be as representative as possible of the population.

Limiting factors came from the self-reported nature of our data collection. Aside from a mandatory psychotic disorder clinical diagnosis, participants self-reported on the presence of their physical and psychological multimorbidity. Although participants were specifically asked if they had been diagnosed by a healthcare professional, it could possibly have led to the underreporting and overreporting of some conditions. Some sensible topics, such as substance use or past suicidal behaviors, could also be related to a social desirability bias. Moreover, the cross-sectional design of the study prevents causal inferences. Additionally, the fact that all participants were recruited from the same emergency department can complicate the generalization of our results to the broader population. Further, participants from the Signature Biobank are not necessarily representative of all people with psychotic disorders, and a previous study showed that participants from the biobank have specific features, including more adversity.^46^ Also, we cannot exclude a potential collider bias in our results due to the study sample. Lastly, we could not include covariates such as medication and household income to account for their potential variance.

The present study examined the association between suicidal behaviors and multimorbidity among patients with a psychotic disorder, addressing an important public health issue that warrants further investigation. Our findings highlight the relevance of considering physical conditions as key risk factors for suicidal behaviors in this population, although the underlying mechanisms require additional study. Given that psychiatric hospitalization remains a critical factor in suicidal behaviors,^47^ individuals with multimorbidity should be closely monitored by clinicians.

Moreover, strategies aimed at preventing the onset of physical conditions in people with psychotic disorders could represent a potential avenue for reducing suicidal behaviors. For instance, promoting physical activity may serve as a low-resource intervention to prevent suicidal ideation and behaviors, although results to date remain heterogeneous.^48^^,^^49^

Results showed important associations between multimorbidity and suicide-related risk such as suicidal ideation among individuals with psychotic disorders. These findings highlight the necessity to further explore interventions as preventive measures among this population to lower the prevalence of co-occurring chronic conditions, improve life quality, and minimize the risk of suicidal behaviors or deaths.

## Supplementary Material

Saito_(2025)_Supplementary_Material_sbag089
