# Supplementary material for: Association between Multimorbidity and Suicidal Behaviors among People with Psychotic Disorders
Source: Schizophr Bull. 2026 Oct 30;52(6):sbag089. doi: 10.1093/schbul/sbag089 (PMC13720728; doi:10.1093/schbul/sbag089)
Supplement: Supplementary file 1 [file saito_(2025)_supplementary_material_sbag089.docx]

SUPPLEMENTARY MATERIAL

**Table A.2** Prevalence of physical and mental conditions

|  | Primary analysis  (N = 748) | Secondary analysis (<30 y.o.)  (N = 240) |
| --- | --- | --- |
| Physical conditions N, %  Arthritis  Asthma  Backpain  Hypertension  Migraine  Bronchitis  Emphysema  COPD  Diabetes (type 2)  Cardiopathy  Cancer  Ulcer  Stroke  Incontinence  Gut disorders  Alzheimer  Epilepsy | 93 (12.43)  100 (13.37)  244 (32.62)  105 (14.04)  195 (26.07)  64 (8.56)  25 (3.34)  25 (3.34)  84 (11.23)  41 (5.48)  16 (2.14)  56 (7.49)  20 (2.67)  77 (10.29)  51 (6.82)  14 (1.87)  26 (3.48) | 15 (6.25)  28 (11.67)  65 (27.08)  19 (7.92)  66 (27.50)  13 (5.42)  3 (1.25)  3 (1.25)  13 (5.42)  8 (3.33)  6 (2.50)  17 (7.08)  7 (2.92)  12 (5.00)  12 (5.00)  -  11 (4.58) |
| Psychological conditions N, %  Mood disorder  Anxiety disorder  Personality disorder  Substance use disorder | 259 (34.63)  244 (32.62)  53 (7.86)  68 (9.09) | 79 (32.92)  85 (35.42)  22 (9.17)  25 (10.42) |

Notes: COPD: Chronic obstructive pulmonary disease ;

**Supplementary file 2:** Results of the association between physical multimorbidity and suicidal ideation - (PHQ-9)

| (N=748) | OR | CI 95% | p-value |
| --- | --- | --- | --- |
| Model 1  Physical multimorbidity  Age  Sex  Unemployed  Model 2  Physical multimorbidity  Age  Sex  Unemployed  Psychotic symptoms  Model 3  Physical multimorbidity  Age  Sex  Unemployed  Psychotic symptoms  AUDIT  DAST-10 | 2.15  0.99  1.16  0.98  2.11  0.99  1.16  0.92  1.40  1.95  0.99  0.97  0.93  1.37  1.04  1.04 | 1.51-3.06  0.98-1.00  0.80-1.69  0.66-1.48  1.47-3.05  0.97-1.00  0.79-1.70  0.61-1.39  1.23-1.60  1.35-2.83  0.98-1.01  0.65-1.45  0.62-1.43  1.20-1.57  1.01-1.06  0.97-1.12 | <0.001*  0.06  0.4  0.9  <0.001*  0.03*  0.5  0.7  <0.001*  <0.001*  0.3  0.9  0.8  <0.001*  0.004*  0.3 |

**Supplementary file 3:** Results of the association between physical multimorbidity and suicidal behaviors - (SBQ-R)

| (N=748) | OR | CI 95% | p-value |
| --- | --- | --- | --- |
| Model 1  Physical multimorbidity  Age  Sex  Unemployed  Model 2  Physical multimorbidity  Age  Sex  Unemployed  Psychotic symptoms  Model 3  Physical multimorbidity  Age  Sex  Unemployed  Psychotic symptoms  AUDIT  DAST-10 | 2.21  0.98  1.77  1.12  2.08  0.98  1.82  1.07  1.57  1.86  0.99  1.45  1.06  1.51  1.05  1.07 | 1.39-3.54  0.96-1.00  1.04-3.10  0.66-1.94  1.28-3.39  0.96-1.00  1.04-3.27  0.62-1.88  1.32-1.89  1.13-3.08  0.97-1.01  0.81-2.66  0.60-1.89  1.26-1.82  1.02-1.09  0.97-1.19 | <0.001*  0.02  0.04  0.7  0.003*  0.017*  0.04*  0.8  <0.001*  0.016*  0.3  0.2  0.8  <0.001*  0.002*  0.2 |

**Supplementary file 4:** Association between multimorbidity and suicidal ideation (PHQ-9) in young adults (<30 years old) with psychosis

| (N=240) | OR | CI 95% | p-value |
| --- | --- | --- | --- |
| Model 1  Physical multimorbidity  Age  Sex  Unemployed  Model 2  Physical multimorbidity  Age  Sex  Unemployed  Psychotic symptoms  Model 3  Physical multimorbidity  Age  Sex  Unemployed  Psychotic symptoms  AUDIT  DAST-10 | 2.62  1.02  1.74  1.12  2.57  1.02  1.75  0.86  1.78  2.33  1.01  1.56  0.85  1.73  1.02  1.03 | 1.43-4.85  0.94-1.11  0.83-3.87  0.58-2.23  1.35-4.96  0.93-1.11  0.79-4.09  0.42-1.77  1.35-2.37  1.19-4.56  0.92-1.11  0.69-3.71  0.42-1.76  1.32-2.32  0.98-1.07  0.91-1.17 | 0.002*  0.6  0.2  0.7  0.004*  0.8  0.2  0.7  <0.001*  0.013*  0.8  0.3  0.7  <0.001*  0.3  0.6 |

**Supplementary file 5:** Association between multimorbidity and suicidal behaviors (SBQ-R) in young adults (<30 years old) with psychosis

| (N=240) | OR | CI 95% | p-value |
| --- | --- | --- | --- |
| Model 1  Physical multimorbidity  Age  Sex  Unemployed  Model 2  Physical multimorbidity  Age  Sex  Unemployed  Psychotic symptoms  Model 3  Physical multimorbidity  Age  Sex  Unemployed  Psychotic symptoms  AUDIT  DAST-10 | 3.02  1.04  1.24  1.39  2.84  1.05  1.22  0.96  1.86  2.27  1.05  1.02  1.03  1.72  1.06  1.07 | 1.38-6.78  0.93-1.17  0.46-3.58  0.58-3.49  1.22-6.71  0.93-1.20  0.41-3.93  0.37-2.54  1.29-2.77  0.93-5.58  0.93-1.20  0.33-3.43  0.39-2.80  1.18-2.59  1.00-1.12  0.90-1.26 | 0.006*  0.5  0.7  0.5  0.016*  0.4  0.7  0.9  0.001*  0.07  0.4  0.9  0.9  0.006*  0.057  0.5 |

**Supplementary file 6**

Notes: The solid line represents the estimated association between multimorbidity and suicidal ideation from the restricted cubic spline model adjusted for covariates, and the shaded area indicates the 95% confidence intervals.

**Supplementary file 7**

Notes: The solid line represents the estimated association between multimorbidity and suicidal ideation from the restricted cubic spline model adjusted for covariates, and the shaded area indicates the 95% confidence intervals.

**Supplementary file 8**

Notes: The solid line represents the estimated association between multimorbidity and suicidal behavior from the restricted cubic spline model adjusted for covariates, and the shaded area indicates the 95% confidence intervals.

**Supplementary file 9**

Notes: The solid line represents the estimated association between multimorbidity and suicidal behavior from the restricted cubic spline model adjusted for covariates, and the shaded area indicates the 95% confidence intervals.
